# Effect of fire needle combined with traditional Chinese medicine on psoriasis: A systematic review and meta-analysis

**DOI:** 10.1097/MD.0000000000035832

**Published:** 2024-02-16

**Authors:** Jinglun Xu, Qiujun Zhou, Fan Xie, Yi Cao, Xiaohong Yang, Maocan Tao

**Affiliations:** a Jinhua Fifth Hospital, Jinhua, China; b Department of First Clinical Medical College, Zhejiang Chinese Medical University, Hangzhou, China; c The First Affiliated Hospital of Zhejiang Chinese Medical University (Zhejiang Provincial Hospital of Chinese Medicine), Hangzhou, China.

**Keywords:** efficacy, fire needle, psoriasis, recurrence, systematic review and meta-analysis, traditional Chinese medicine

## Abstract

**Background:**

The mechanism of action of fire acupuncture and Chinese medicine in psoriasis is unclear. In this paper, the efficacy of the 2 therapies was compared through a comprehensive analysis of their recurrence rates for clinical reference.

**Methods:**

In this meta-analysis, we searched PubMed, Embase, Cochrane Library, CNKI, Wanfang, CQVIP, and CBM data from the establishment of the databases to May 2023. The study proposed to use randomized controlled trial research methods, excluding published literature, unpublished literature, literature with incomplete or inadequate information, animal experiments, literature reviews and systematic studies. Data were processed using STATA 15.1 software.

**Results:**

Our group previous study found that the clinical efficacy of the fire-acupuncture group was significantly improved compared to that of Chinese herbal medicine alone (RR = 1.20, 95% CI: 1.13–1.27). Also, there were significant reductions in Psoriasis Area and Severity Index (PASI) score (SMD = −1.04,95% CI: −1.48 to −0.60), area of skin damage (SMD = −0.40,95% CI: −0.75 to −0.04), and pruritus (SMD = −1.04,95% CI: −1.32 to −0.76). Our previous study found that Dermatology Life Quality Index (DLQI) was significantly lower in the fire acupuncture group compared to herbal medicine alone (SMD = −1.61,95% CI: −3.08 to −0.15). The combined analysis found that the recurrence rate was significantly lower in the fire-acupuncture group compared to herbal medicine alone (RR = 0.21,95% CI:0.07–0.60).

**Conclusion:**

Fire needle can improve the efficacy of TCM in the treatment of psoriasis, including the area, severity and itching of skin lesions, and reduce the recurrence rate, which is worthy of clinical promotion.

## 1. Introduction

Psoriasis is a skin disease characterized by a chronic inflammatory response that mainly manifests as abnormal keratinocyte hyperplasia accompanied by the local infiltration of inflammatory cells.^[1]^ Psoriasis can vary in severity, with some individuals experiencing mild symptoms and others experiencing more severe effects. The main manifestations of psoriasis include plaque (psoriasis vulgaris), guttate, inverse, pustular, erythrodermic, and nail psoriasis.^[2]^ Although a global health concern affecting approximately 1% to 3% of the world population, psoriasis is not contagious and is believed to have a genetic predisposition since it often occurs in families.^[3]^ The etiology and pathogenesis of psoriasis are unclear^[4]^; modern medicine is relatively consistent, and the view is related to genetic, immune, metabolic, infection, mental, and other factors.^[5]^ Because of the unique clinical symptoms of psoriasis, traditional Chinese medicine (TCM) believes that psoriasis belongs to the category of “tinea stem” and “tinea pine bark,” which is called “white” in modern TCM.^[6]^

The treatment of psoriasis in Western medicine is still being explored. Currently, there are oral preparations, topical drugs, phototherapy, and biological agents; however, their clinical effects are not ideal.^[7]^ Although treatment can temporarily relieve symptoms, the recurrence rate after drug discontinuation is high. Moreover, drugs are generally expensive and the economic burden caused by long-term or even lifelong treatment seriously affects patients’ daily life, reducing their quality of life, and is not conducive to ensuring patient compliance, thus affecting wide promotion of medicine in clinical practice.^[8]^

Chinese medicine has a long history of treating diseases and offers unique advantages. It focuses on a holistic view that combines evidence differentiation with treatment methods, appropriate coordination of the 3 methods, flexible treatment methods, low side effects, and low recurrence rates after clinical cure.^[9,10]^ Fire needle therapy has the characteristics of simplicity, convenience, testing, and integrity and is an important part of the traditional characteristic therapy of TCM.^[11]^ In ancient times, it was also called “thorn” and “burnt needle,” which specifically refers to the tip of the needle after burning red and quickly stabbed into the acupoint. When the fire needle plays the therapeutic role of acupuncture on the acupoints, it can borrow fire to help Yang, open the door to remove evil spirits, and play the role of running qi and blood, removing blood stasis, activating collaterals, dispelling wind, and relieving itching.^[12]^ The mechanisms of action of fire acupuncture and TCM in psoriasis remain unclear. This project aimed to conduct a meta-analysis of the domestic and international literature on the combination of fire acupuncture and TCM for the treatment of psoriasis, evaluate their efficacy, and analyze their efficacy and recurrence rates to guide clinical practice.

## 2. Methods

### 2.1. Literature review inclusion and exclusion criteria

#### 2.1.1. Inclusion criteria.

1. Subjects: Patients with psoriasis.
2. Combination of fire acupuncture and TCM.
3. Control: TCM alone.
4. Outcome indicators: Total effective rate, Psoriasis Area and Severity Index (PASI), Dermatology Life Quality Index (DLQI), skin damage area, degree of itching, ratio of CD4^+^/CD8^+^, and recurrence rate.
5. Study design: Randomized controlled trial.

#### 2.1.2. Exclusion criteria.

Duplicate publications: Studies for which full text was not available or for which data extraction was not possible, animal studies, reviews, and systematic reviews.

### 2.2. Search strategy

In this meta-analysis, we searched the PubMed, Embase, Cochrane Library, CNKI, Wanfang, CQVIP, and CBM databases from inception to May 2023. The search terms were: (((((fire needle[Title/Abstract]) OR (fire puncture[Title/Abstract])) OR (fire needles[Title/Abstract])) OR (fire punctures[Title/Abstract])) AND ((((((“Psoriasis”[Mesh]) OR (Psoriasis[Title/Abstract])) OR (Pustulosis of Palms[Title/Abstract] AND Soles[Title/Abstract])) OR (Pustulosis Palmaris et Plantaris[Title/Abstract])) OR (Palmoplantaris Pustulosis[Title/Abstract])) OR (Pustular Psoriasis of Palms[Title/Abstract] AND Soles[Title/Abstract]))) AND ((((traditional Chinese medicine[Title/Abstract]) OR (traditional Chinese medicines[Title/Abstract])) OR (Decoction[Title/Abstract])) OR (Recipe[Title/Abstract])).

### 2.3. Literature screening and data extraction

Two researchers searched for, screened, and captured the information. Any questions or disagreements were resolved through consultation with a third party. Collected information included author, year, study design, number of cases, and outcome indicators.

### 2.4. Literature quality assessment

Two researchers conducted separate independent evaluations of the quality of the retrieved studies using the Review Manager 5.3 software risk assessment tool according to random sequence generation, allocation concealment, blinding, whether research results were blinded to review, and completeness of the outcome data using the Cochrane Risk Assessment Scale, sex, selection of reported research outcomes, and other biases. Disagreements were resolved through discussion or consultation with a third party. This meta-analysis was performed in accordance with the relevant items in the Preferred Reporting Items for Systematic Reviews and Meta Analysis (PRISMA) statement.^[13]^

### 2.5. Data synthesis and statistical analysis

The data were analyzed using STATA version 15.1. Standardized mean differences (SMD) (95% confidence interval [CI]) were used as continuous variables. *I*^2^ was used to evaluate cell heterogeneity. If the test for heterogeneity was *P* ≥ .1 and *I*^2^ ≤ 50%, homogeneity between studies was indicated and the studies were analyzed together using a fixed effects model. If *P* < .1 and *I*^2^ > 5%, significant intergroup heterogeneity was indicated; if a difference was noted, its source was identified using a sensitivity analysis. If the differences were still large, a random-effects model was used or the results of the combined study were discarded in favor of the descriptive analysis. Publication bias was analyzed using funnel graphs and Egger tests.

## 3. Results

### 3.1. Literature search results

**Figure 1.**
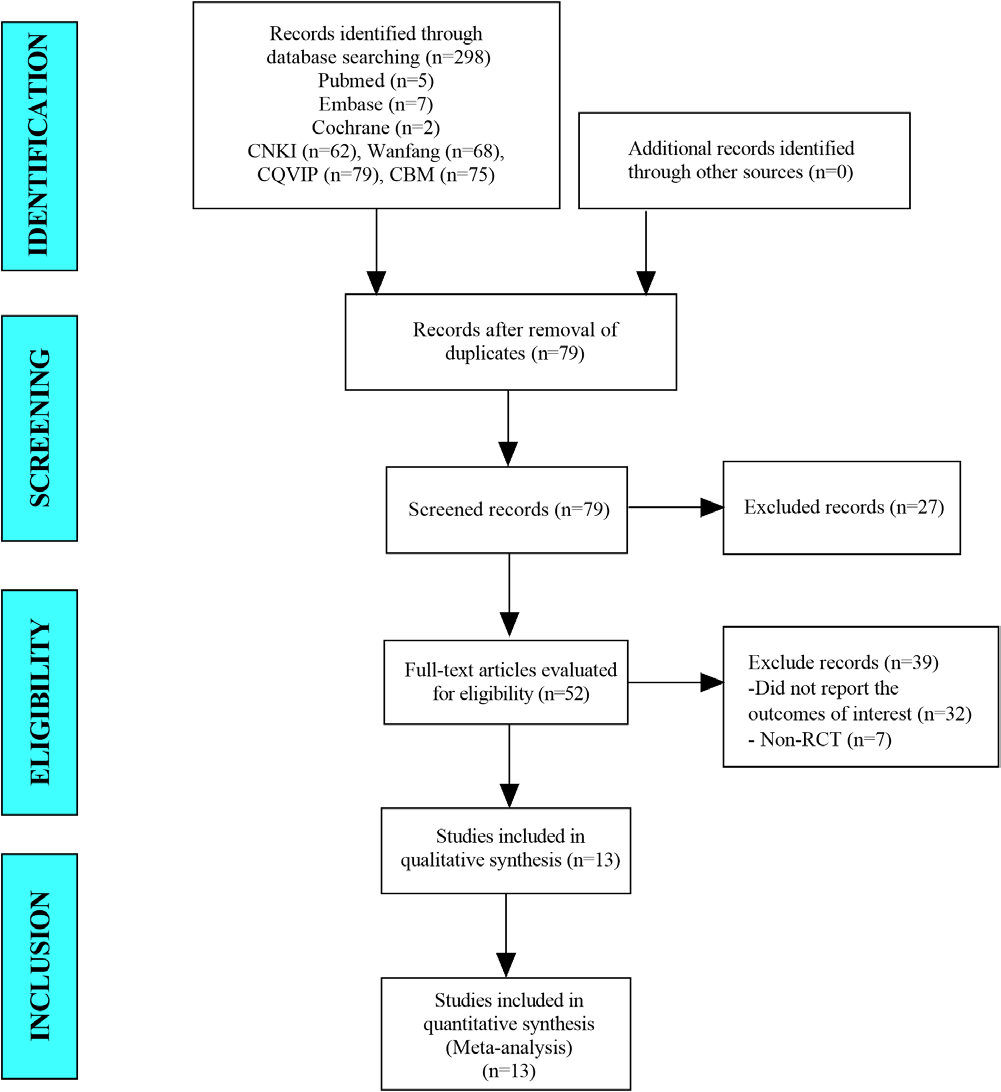
Flow diagram of the study selection process.

A total of 298 studies were collected from the literature for this study. After the exclusion of duplicate studies, 79 studies were included. A total of 52 papers remained after the title and abstract screening. Ultimately, 13 studies were included in the meta-analysis (Fig. 1).

### 3.2. Baseline characteristics and quality assessment of included studies

#### 3.2.1. Baseline characteristics.

This meta-analysis included 13 randomized controlled trial studies. The sample size was 48 to 122, with a total of 1088 patients (543 in the herbal medicine group, 545 in the Chinese medicine group). The mean patient age in the combination therapy and Chinese herbal medicine groups ranged from 33.54 to 48.57 and 32.95 to 49.87 years, respectively; the ages of the 2 groups were comparable. The primary Chinese medicines used were Huoxue Jiedu, Liangxue Xiaofeng San, Xuefu Zhuyu, Huoxue Zhuyu, Taohong Siwu, Huoxue Sanyu Xiaoyin, and Wugong Baidu (Table 1).

**Table 1:** The baseline characteristics of the included studies.

Author	Yr	Study design	Sample size		Sex (male/female)		Age		Duration		Measurements	
			Combination treatment group	TCM group	Combination treatment group	TCM group	Combination treatment group	TCM group	Combination treatment group	TCM group	Combination treatment group	TCM group
Li RT^[14]^	2016	RCT	25	23	18/7	15/8	43.28 ± 12.76	45.04 ± 13.65	15.12 ± 9.52	12.70 ± 9.96	Fire needle plus Huoxue Jiedu Decoction	Huoxue Jiedu Decoction
Dun G^[15]^	2017	RCT	61	61	34/27	38/23	35.49 ± 10.38	36.21 ± 10.32	6.67 ± 2.14	7.06 ± 2.26	Fire needle plus Liangxue Xiaofeng San	Liangxue Xiaofeng San
Dong LL^[16]^	2018	RCT	45	45	27/18	29/16	35.80 ± 5.20	36.40 ± 5.60	5.30 ± 3.00	5.00 ± 3.30	Fire needle plus Liangxue Xiaofeng San	Liangxue Xiaofeng San
Li WJ^[17]^	2019	RCT	31	31	15/16	16/15	36.90 ± 10.76	37.06 ± 11.92	6.45 ± 3.20	7.29 ± 3.37	Fire needle plus Xuefu Zhuyu Decoction	Xuefu Zhuyu Decoction
Deng JL^[18]^	2019	RCT	53	53	31/22	30/23	33.54 ± 7.91	32.95 ± 8.12	7.25 ± 6.76	7.36 ± 6.84	Fire needle plus Huoxue Jiedu Decoction	Huoxue Jiedu Decoction
Wu KJ^[19]^	2019	RCT	43	43	22/21	23/20	38.78 ± 3.59	38.91 ± 3.67	/	/	Fire needle plus Huoxue Jiedu Decoction	Huoxue Jiedu Decoction
Wang H^[20]^	2020	RCT	55	55	66/44		47.2 ± 11.6		/	/	Fire needle plus Huoxue Jiedu Decoction	Huoxue Jiedu Decoction
Gao Y^[21]^	2020	RCT	39	39	23/16	22/17	48.34 ± 5.84	49.87 ± 5.65	12.29 ± 6.83	10.46 ± 5.71	Fire needle plus Huoxue Jiedu Decoction	Huoxue Jiedu Decoction
Zhu J^[22]^	2020	RCT	60	60	32/28	29/31	48.57 ± 4.09	49.56 ± 4.17	9.11 ± 1.28	6.50 ± 1.31	Fire needle plus Huoxue Zhuyu Decoction	Huoxue Zhuyu Decoction
Li YY^[23]^	2021	RCT	23	24	11/12	13/11	39.87 ± 11.56	40.25 ± 11.78	10.87 ± 5.16	11.29 ± 6.10	Fire needle plus Taohong Siwu Decoction	Taohong Siwu Decoction
Liu QY^[24]^	2022	RCT	30	29	18/12	16/13	41.13 ± 5.23	40.69 ± 4.97	4.07 ± 1.49	4.59 ± 1.15	Fire needle plus Huoxue Sanyu Xiaoyin Decoction	Huoxue Sanyu Xiaoyin Decoction
Xiao X^[24]^	2022	RCT	31	35	14/17	16/19	37.16 ± 11.09	35.40 ± 10.09	3.45 ± 1.27	3.76 ± 1.14	Fire needle plus Wugong Baidu Decoction	Wugong Baidu Decoction
Meng R^[25]^	2022	RCT	47	47	28/19	26/21	45.31 ± 8.25	44.64 ± 8.32	1.71 ± 0.57	1.57 ± 0.52	Fire needle plus Huoxue Sanyu Decoction	Huoxue Sanyu Decoction

RCT = randomized controlled trial, TCM = traditional Chinese medicine.

#### 3.2.2. Quality assessment of included studies.

**Figure 2.**
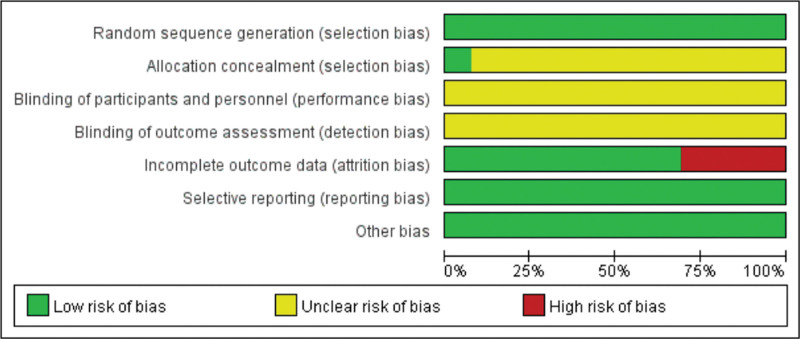
Risk of bias items.

**Figure 3.**
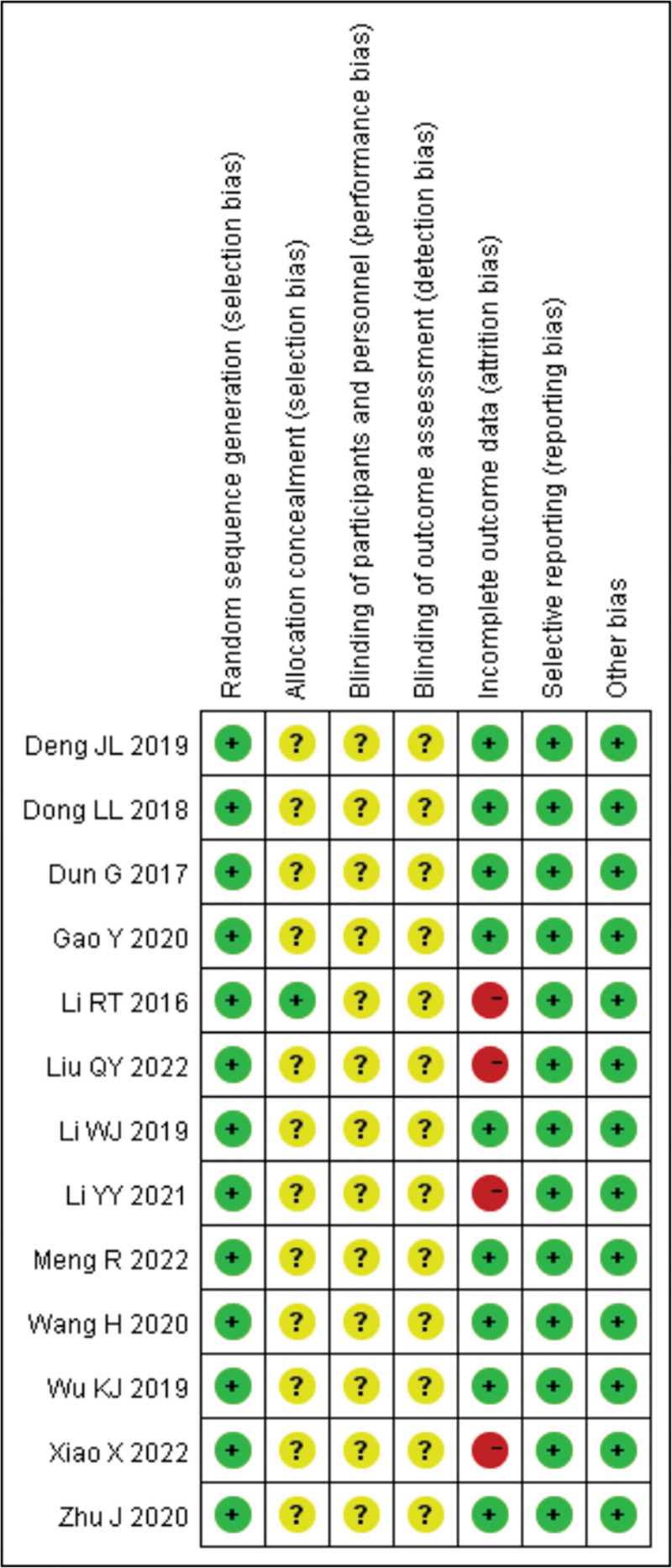
Risk of bias summary.

Method quality was evaluated according to the PRISMA guidelines. Thirteen papers described randomization, while only 1 paper described hidden assignment. No blinding was performed. Only 3 studies were lost to follow-up (Figs. 2–3).

### 3.3. Meta-analysis results

#### 3.3.1. Total effective rate.

**Figure 4.**
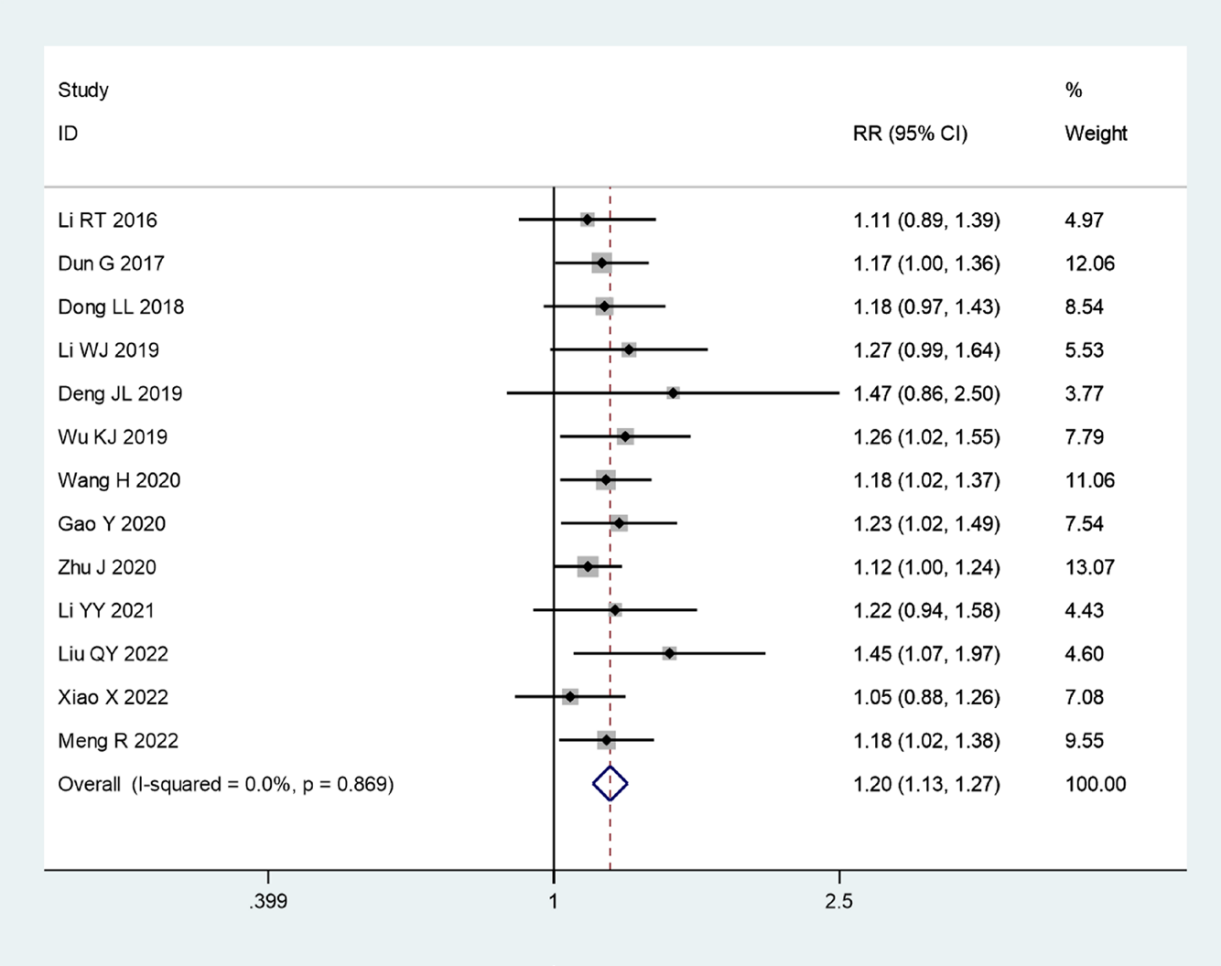
Forest plot comparing the total effective rate of fire needle plus traditional Chinese medicine (TCM) with TCM alone in the treatment of psoriasis.

Thirteen reports compared the efficacy of fire acupuncture combined with TCM in psoriasis. As there was no significant heterogeneity (*I*^2^ = 0.0%, *P* = .869), a fixed-effects model was used for the meta-analysis. We previously found that the overall effective rate was significantly higher in the fire acupuncture versus TCM group (relative risk, 1.20; 95% CI, 1.13–1.27; *P* = .000) (Fig. 4).

#### 3.3.2. PASI score

**Figure 5.**
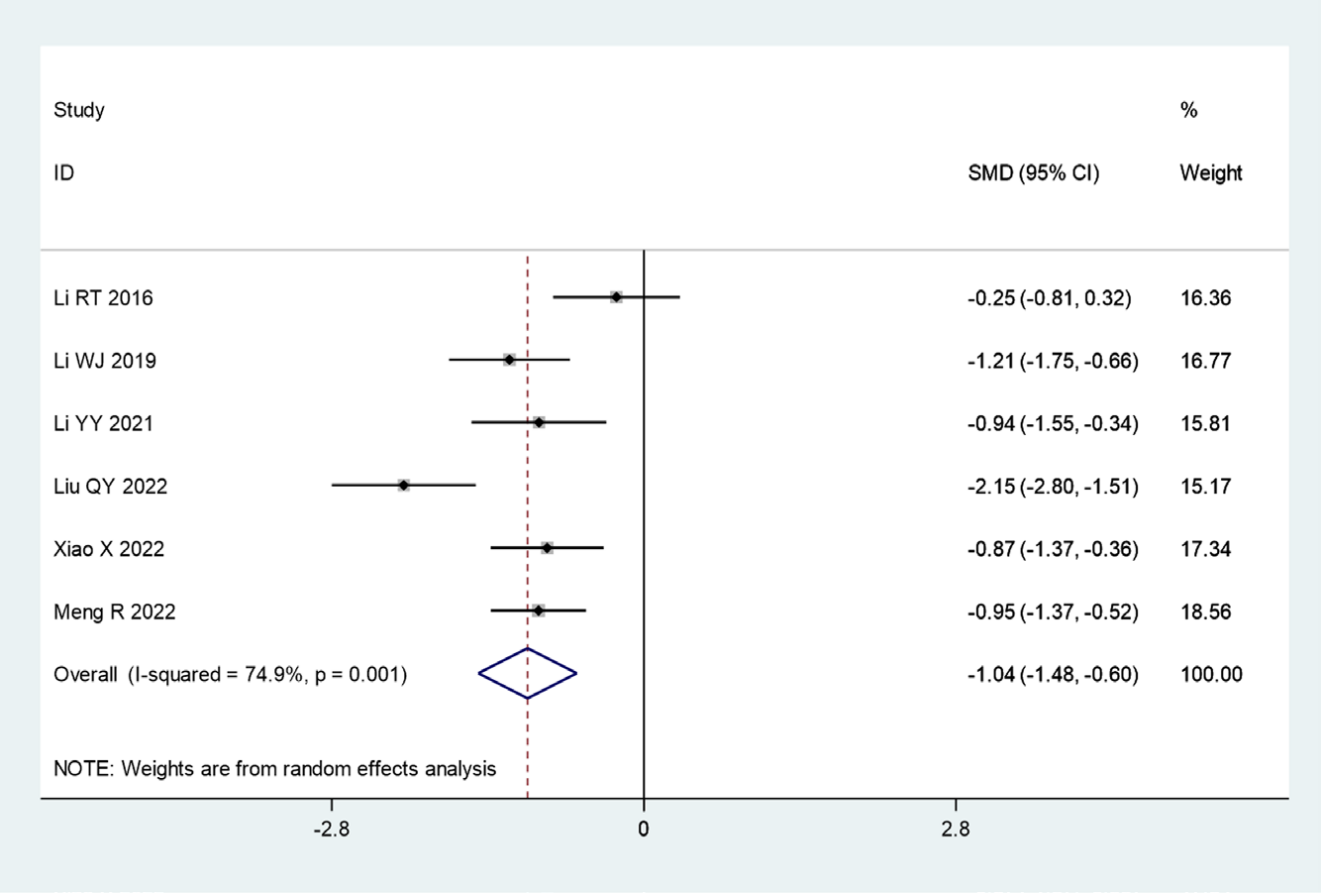
Forest plot comparing the mean Psoriasis Area and Severity Index score of the fire needle combined with TCM with the TCM alone group in the treatment of psoriasis. SMD = standardized mean differences; TCM = traditional Chinese medicine.

Six studies compared the PASI scores of fire acupuncture plus TCM versus TCM alone. Owing to significant heterogeneity (*I*^2^ = 74.9%, *P* = .001), a random-effects model was used for the meta-analysis. The combined analysis found that the mean PASI score was significantly lower in the fire acupuncture plus TCM group versus the TCM alone group (SMD, −1.04; 95% CI, −1.48 to −0.60) (Fig. 5).

#### 3.3.3. Skin damage area

**Figure 6.**
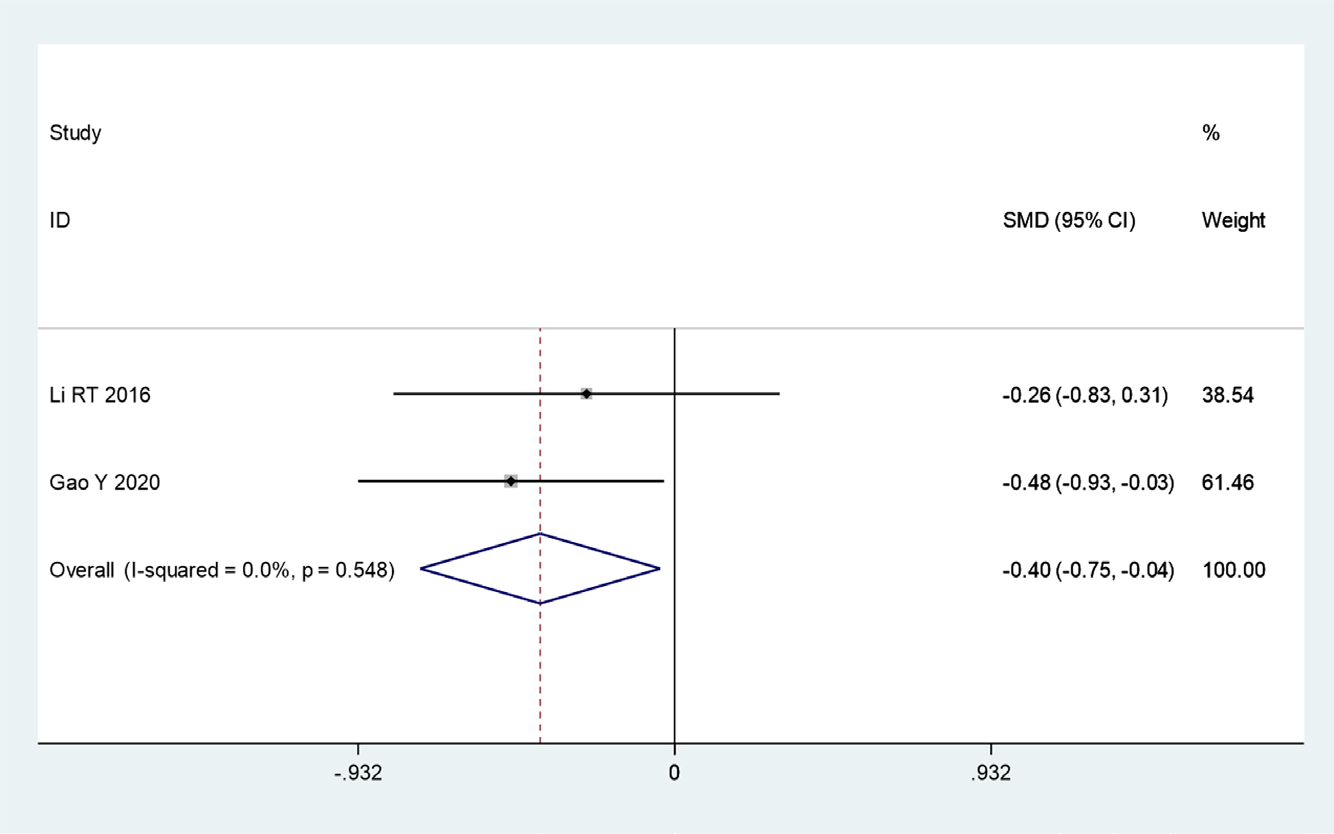
Forest plot comparing the skin damage area of fire needle combined with TCM with TCM alone in the treatment of psoriasis (after sensitivity analysis). SMD = standardized mean differences; TCM = traditional Chinese medicine.

This study reports 3 clinical trials comparing the effects of fire acupuncture plus TCM versus TCM alone on psoriatic lesions. The sensitivity analysis (*I*^2^ = 74.9%, *P* = .001) showed that the results were greatly influenced by those of the study by Zhu et al Excluding this test significantly reduced the difference (*I*^2^ = 0, *P* = .548). A meta-analysis was conducted using a fixed-effects model. The combined analysis revealed that the area of skin damage was smaller in the fire acupuncture plus TCM group than in the TCM group (Fig. S1, http://links.lww.com/MD/L672, Fig. 6).

#### 3.3.4. Degree of itching

**Figure 7.**
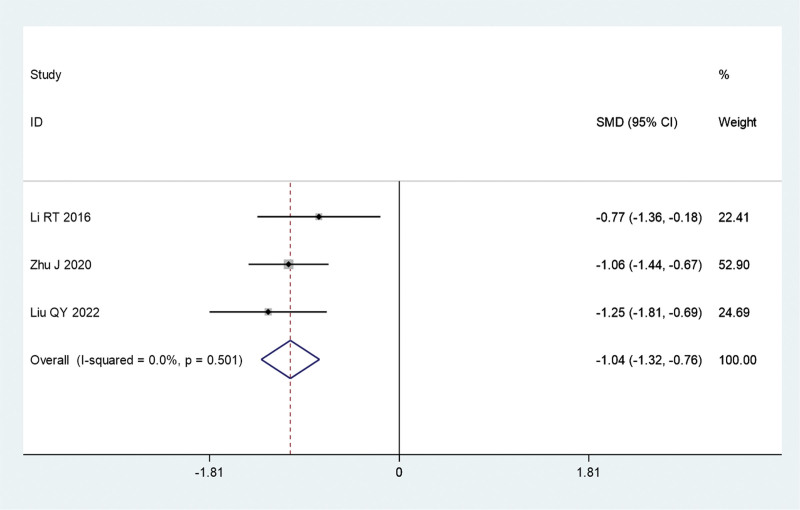
Forest plot comparing the degree of itching of fire needle combined with TCM with TCM alone in the treatment of psoriasis (after sensitivity analysis). SMD = standardized mean differences; TCM = traditional Chinese medicine.

Four reports compared the application of fire acupuncture plus TCM versus TCM alone. A sensitivity analysis revealed that the results from the study by Dun et al significantly influenced those of the experiment. This difference was significantly reduced after the exclusion of this trial (*I*^2^ = 0, *P* = .501). A meta-analysis was conducted using a fixed-effects model. The combined analysis revealed that the fire acupuncture plus TCM group had better clinical efficacy than the TCM alone group (SMD, −1.04; 95% CI, −1.32 to −0.76; *P* = .000) (Fig. S2, http://links.lww.com/MD/L673, Fig. 7).

#### 3.3.5. Dermatology life quality index

**Figure 8.**
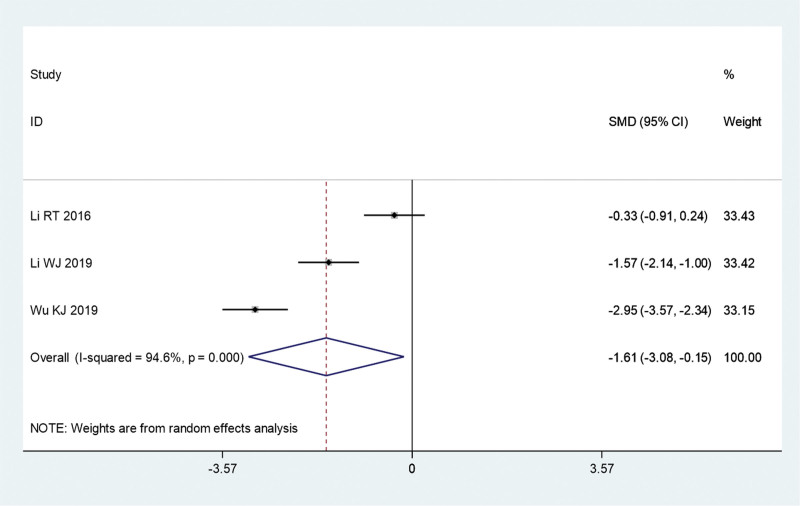
Forest plot comparing the Dermatology Life Quality Index score of fire needle combined with TCM with TCM alone in the treatment of psoriasis. SMD = standardized mean differences; TCM = traditional Chinese medicine.

Three clinical trials were conducted to compare the DLQI scores of the fire acupuncture plus TCM group versus TCM alone group. Because of the significant heterogeneity (*I*^2^ = 94.6%, *P* = .000), a random-effects model was used for the meta-analysis. The combined study found significantly lower DLQI scores in the fire acupuncture plus TCM group compared to the TCM alone group (SMD, −1.61; 95% CI, −3.08 to −0.15; *P* = .030) (Fig. 8).

#### 3.3.6. Ratio of CD4^+^/CD8^+^ cells

**Figure 9.**
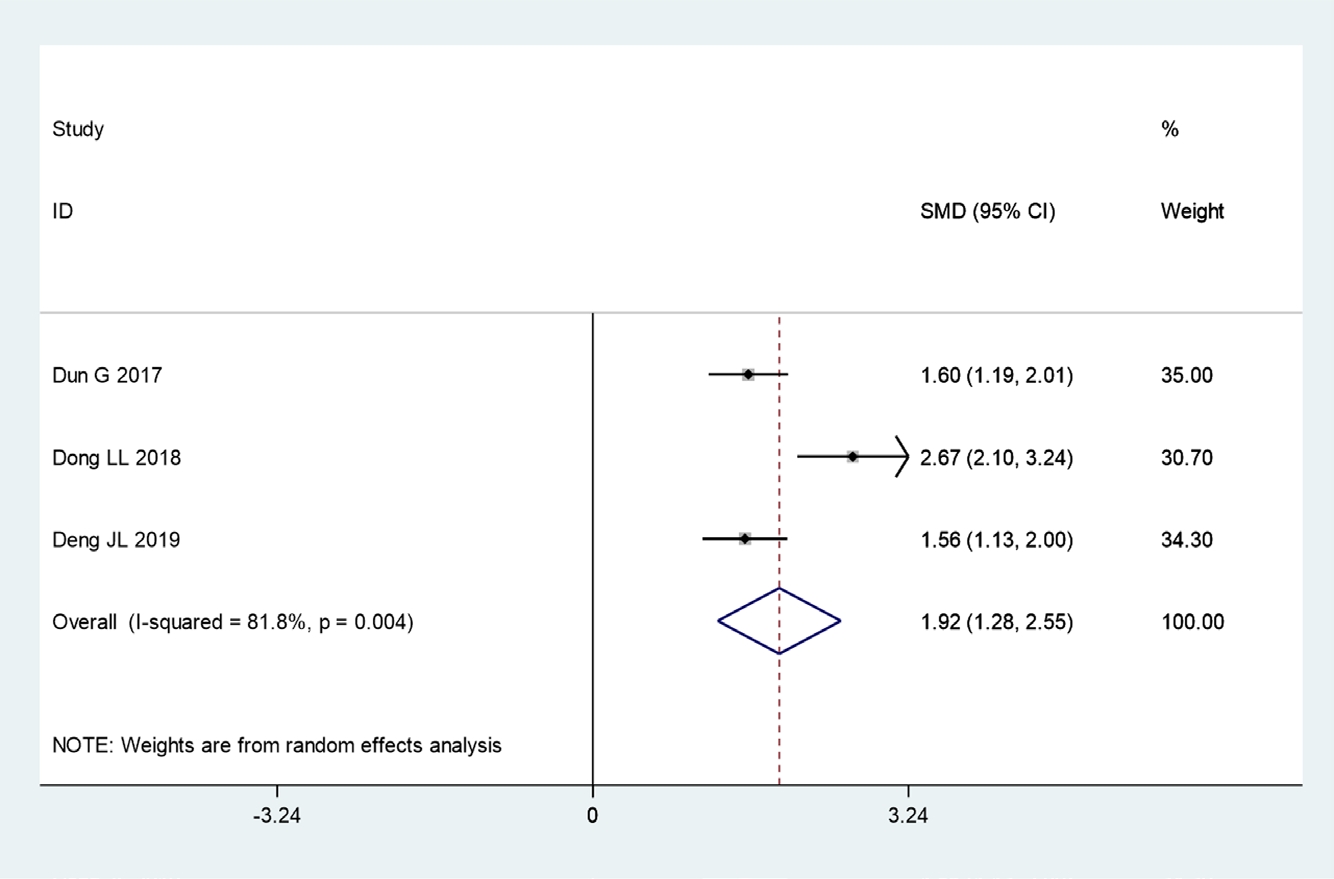
Forest plot comparing the ratio of CD4^+^/CD8^+^ cells of fire needle combined with TCM with TCM alone in the treatment of psoriasis. SMD = standardized mean differences; TCM = traditional Chinese medicine.

There are 3 such reports in the literature. We compared the effects of fire acupuncture plus TCM versus TCM alone on the CD4^+^/CD8^+^ lymphocyte ratio in the peripheral blood of patients with psoriasis. Due to the significant heterogeneity (*I*^2^ = 81.8%, *P* = .004), a random-effects model was used for the meta-analysis. Our previous study found that the CD4^+^/CD8^+^ cell ratio was significantly higher in the fire acupuncture plus TCM group than in the TCM alone group (SMD, 1.92; 95% CI, 1.28–2.55; *P* = .000) (Fig. 9).

#### 3.3.7. Recurrence rate

**Figure 10.**
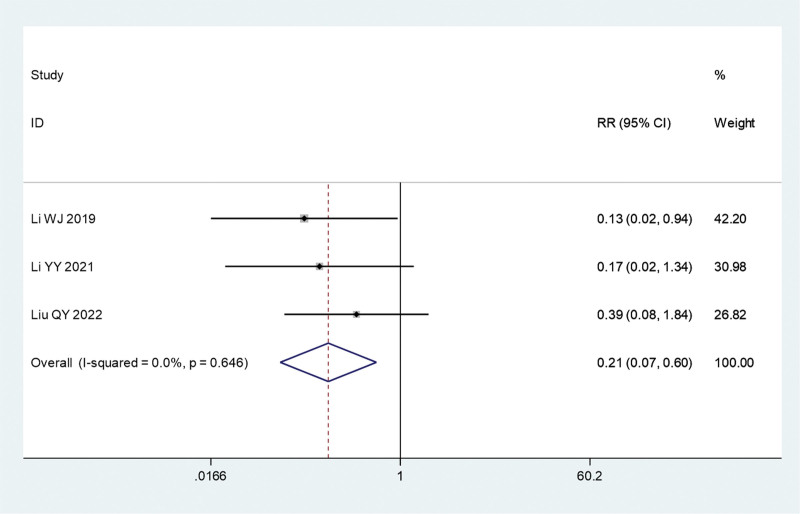
Forest plot comparing the recurrence rate of fire needle combined with TCM with TCM alone in the treatment of psoriasis. CI = confidence interval, RR = relative risk, TCM = traditional Chinese medicine.

This paper reports 3 different clinical trials comparing fire acupuncture plus TCM and TCM alone. As no significant heterogeneity was noted (*I*^2^ = 0.0%, *P* = .646), a fixed-effects model was used for the meta-analysis. Our previous study found a significantly lower recurrence rate in the fire acupuncture plus TCM group than in the TCM alone group (relative risk, 0.21; 95% CI, 0.07–0.60; *P* = .003) (Fig. 10).

#### 3.3.8. Publication bias

**Figure 11.**
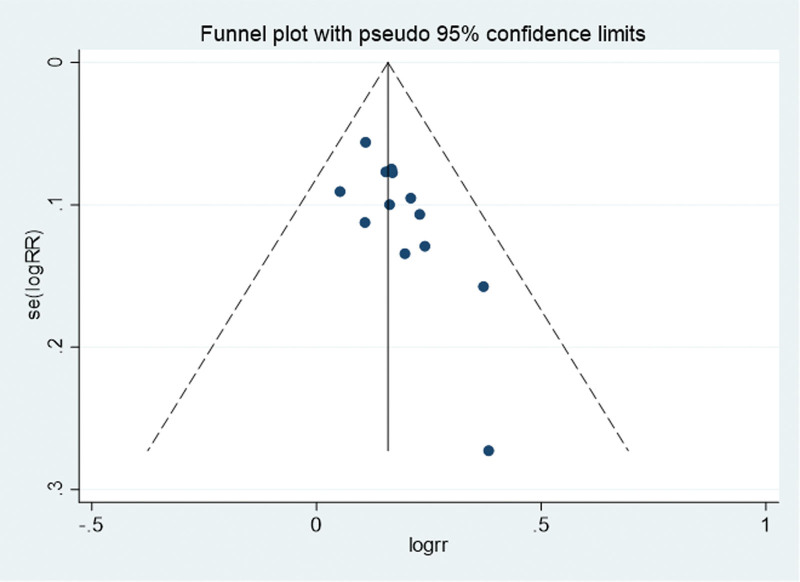
Funnel plot used to assess publication bias.

Figure 11 shows the funnel plot constructed in this study, which was not perfectly symmetrical. Egger test based on the funnel plot had *P* = .011, suggesting that the study may have been biased by publication (Fig. 11).

#### 3.3.9. Sensitivity analysis

The sensitivity analysis excluded each included test individually and then summed the remaining tests to evaluate the effect of a single test on the overall meta-analysis. The results of the sensitivity analyses performed in this study are shown in Figures S3–9; http://links.lww.com/MD/L674; http://links.lww.com/MD/L675; http://links.lww.com/MD/L676; http://links.lww.com/MD/L677; http://links.lww.com/MD/L678; http://links.lww.com/MD/L679; http://links.lww.com/MD/L680.

## 4. Discussion

Psoriasis was first recorded during the Sui Dynasty in China. *On the Etiology of psoriasis · Tinea psoriasis* stated, “Tinea psoriasis has Kuang Guo, the skin is dry and the rope is itching, and the white dust is also scratched out.” With the aggravation of air pollution and increase in social pressure, the incidence of silver dust disease is increasing annually. Western medicines primarily use glucokinins, immunosuppressants, and immunomodulators. Although they can temporarily relieve the disease, their use is associated with a high recurrence rate and long-term adverse effects. However, TCM features fewer toxic effects. We previously reviewed 13 studies on the treatment of psoriasis with fire acupuncture plus TCM in 1088 cases and compared the total effective rate, PASI score, DLQI score, extent of lesion area, presence of pruritus, CD4^+^/CD8^+^ ratio, and relapse rate.

First, a comparison of the 2 groups of patients to determine the difference in efficacy during treatment suggested that fire needle therapy can significantly improve the efficacy of TCM for psoriasis. Notably, a previously published meta-analysis showed that fire needles combined with oral medicines were effective at treating psoriasis. This study further supports that fire needle therapy combined with TCM is effective in the treatment of psoriasis.^[26]^ In addition, the PASI score and skin damage area were significantly reduced after fire needle therapy combined with TCM. Fire needles can regulate the stress response of the skin, enhance the phagocytic function of white blood cells, and affect relevant T cell factors, thereby reducing local inflammatory edema, promoting inflammatory absorption, and relieving inflammation.^[27]^ In addition, fire needles can regulate the central nervous system under the cerebral cortex, specifically suppressing the itching sensation by exciting pain in the sensory center.^[28]^ This finding is in line with the results of our previous study, which showed a greater difference in the fire acupuncture group than in the TCM group. In terms of quality of life, the DLQI score was significantly lower in the fire acupuncture group than in the TCM alone group. This indicates that fire acupuncture plus TCM has better efficacy and contributes more to patient satisfaction than TCM alone.

Psoriasis is a chronic inflammatory skin disease with abnormal immune function associated with disorders in T-cell regulation.^[29]^ Patients with psoriasis have much higher levels of cytokines that mediate immunity in patients with psoriasis, including tumor necrosis factor-α,^[30]^ interleukin-1,^[31]^ and interleukin-6,^[32]^ which mediate innate immunity. In the present study, the pooled results showed that the ratio of CD4^+^/CD8^+^ cells was significantly higher in the fire needle therapy combined with TCM group than in the TCM alone group. This indicates that the fire needle treatment further increased the infiltration level of CD4^+^ T cells. Thus, TCM drugs that mainly improve the differentiation of CD4^+^ T cells may be the first choice for the treatment of psoriasis.

This study also explored whether combination therapy has an advantage in terms of recurrence rate. A comprehensive analysis revealed that the recurrence rate was significantly lower in the fire acupuncture plus TCM group than in the TCM alone group. This study also provides a basis for clinicians to choose a combination of fire acupuncture and TCM for the treatment of psoriasis.

This meta-analysis has the following shortcomings. First, there was considerable heterogeneity in the analysis of the DLQI score and ratio of CD4^+^/CD8^+^ cells, which reduced our confidence about its results; heterogeneity may be related to patient status and drug use, but the number of existing studies and descriptions of patient characteristics were insufficient to support subgroup analyses. Second, the presence of publication bias hindered the study objectivity. Therefore, future studies are required to further substantiate the findings of this study and avoid publication bias.

## 5. Conclusion

Fire needles can improve the efficacy of TCM in the treatment of psoriasis, including extent of lesion area, severity, and itching, and reduce its recurrence rate, making it worthy of clinical promotion.

## Author contributions

**Conceptualization:** Qiujun Zhou, Maocan Tao.

**Data curation:** Qiujun Zhou.

**Formal analysis:** Fan Xie.

**Funding acquisition:** Fan Xie, Yi Cao, Xiaohong Yang.

**Methodology:** Qiujun Zhou, Xiaohong Yang.

**Software:** Qiujun Zhou.

**Validation:** Yi Cao.

**Writing – original draft:** Jinglun Xu, Qiujun Zhou.

**Writing – review & editing:** Jinglun Xu, Qiujun Zhou, Maocan Tao.

## Supplementary Material


















