# Supplementary figures and images for: Effect of fire needle combined with traditional Chinese medicine on psoriasis: A systematic review and meta-analysis
Source: Medicine (Baltimore). 2024 Feb 16;103(7):e35832. doi: 10.1097/MD.0000000000035832 (PMC10869083; doi:10.1097/MD.0000000000035832)

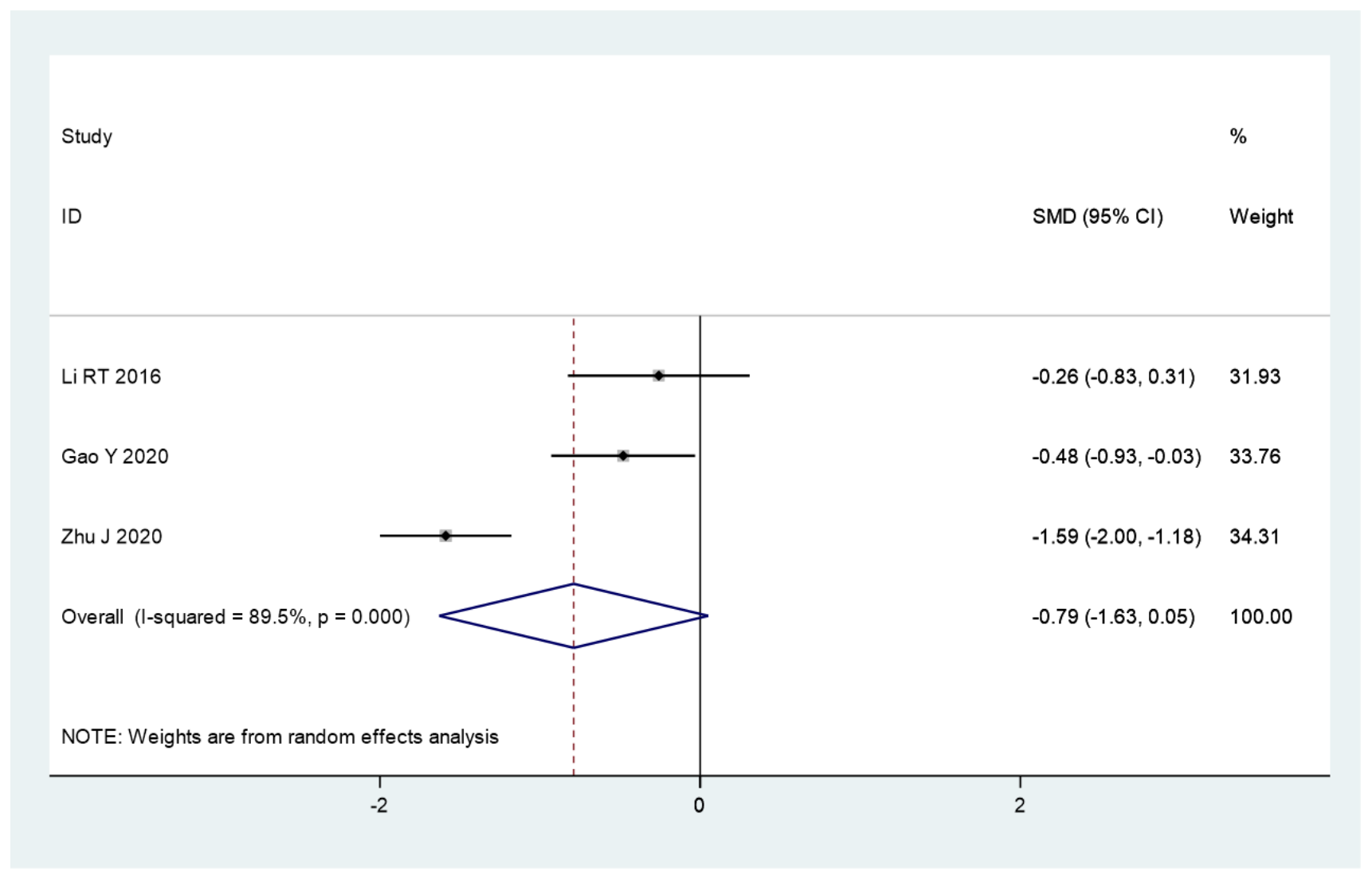

Supplement: Supplementary file 1 [file medi-103-e35832-s001.tif]

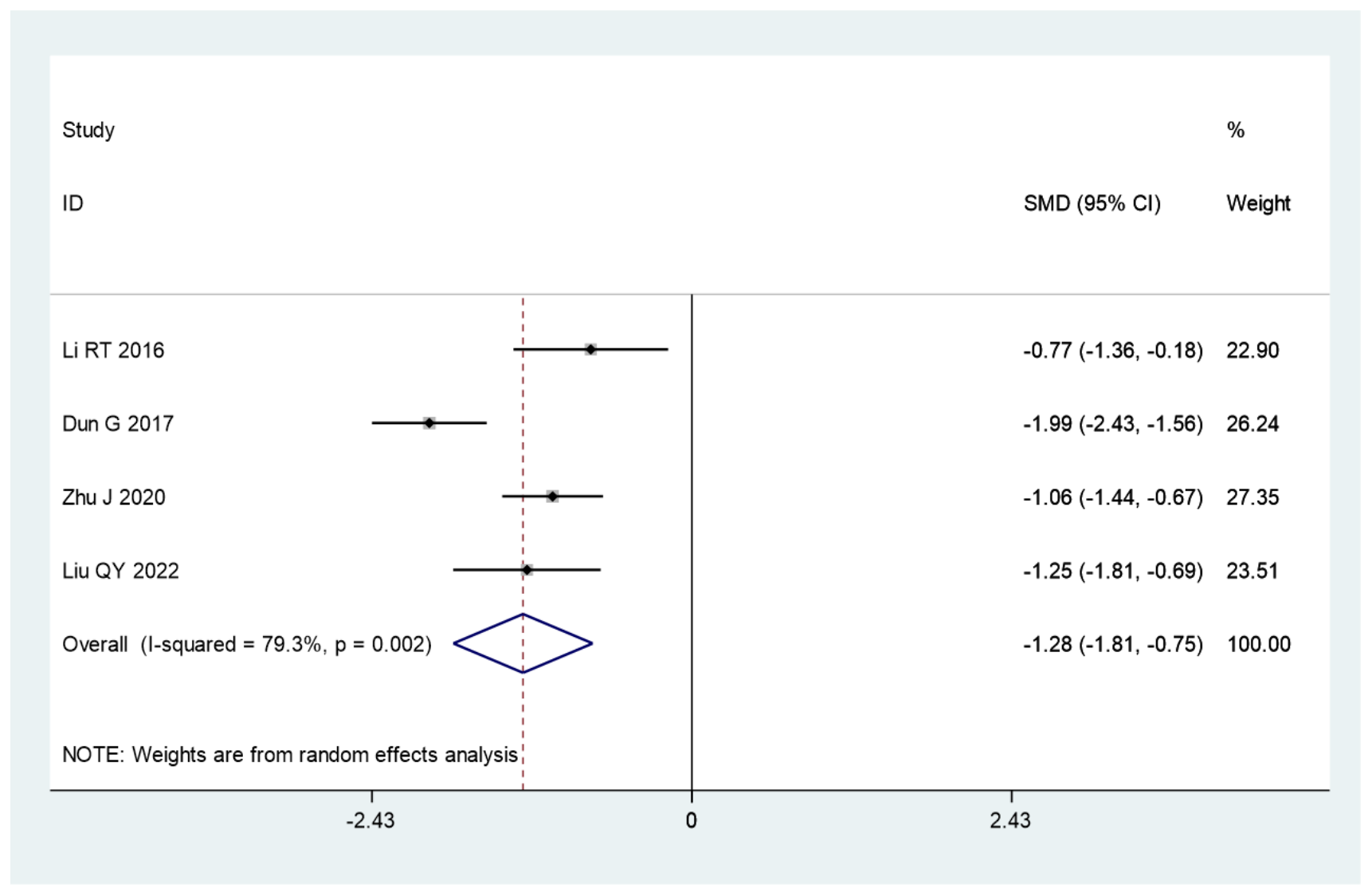

Supplement: Supplementary file 2 [file medi-103-e35832-s002.tif]

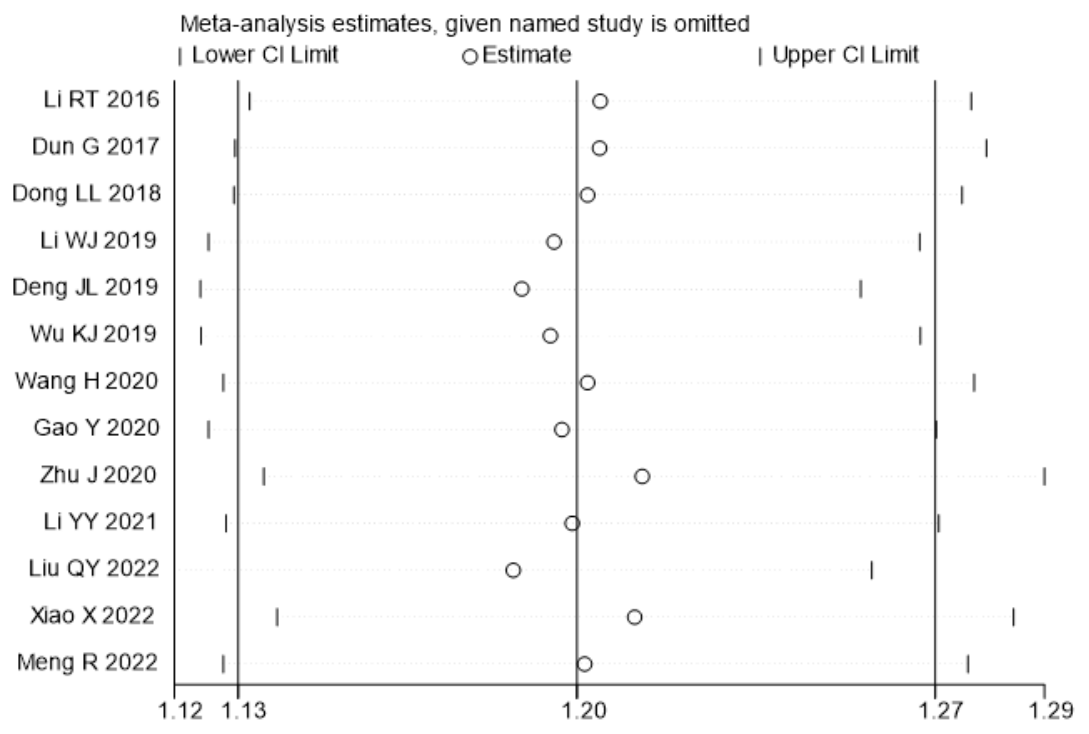

Supplement: Supplementary file 3 [file medi-103-e35832-s003.tif]

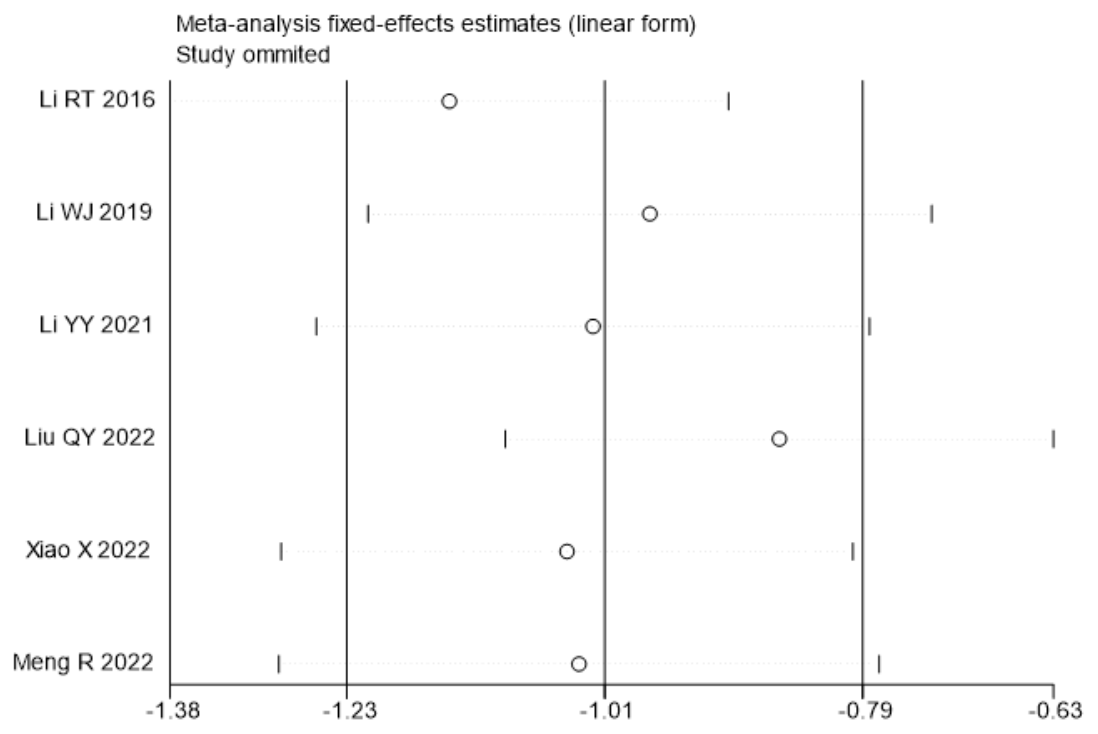

Supplement: Supplementary file 4 [file medi-103-e35832-s004.tif]

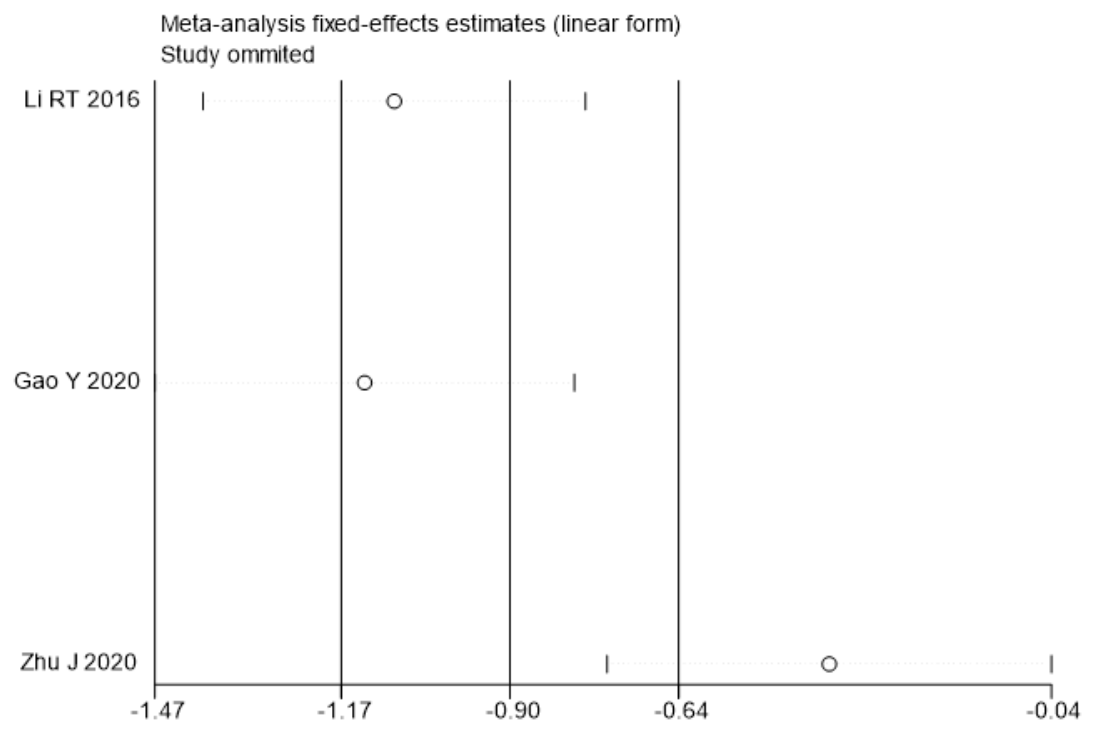

Supplement: Supplementary file 5 [file medi-103-e35832-s005.tif]

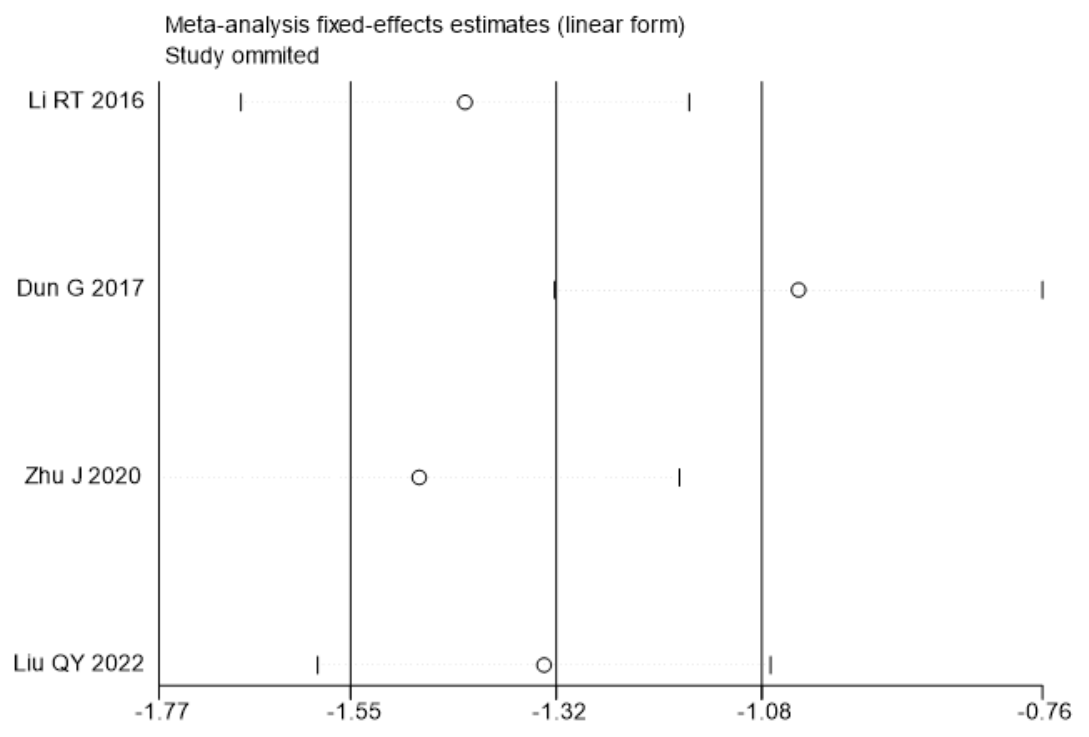

Supplement: Supplementary file 6 [file medi-103-e35832-s006.tif]

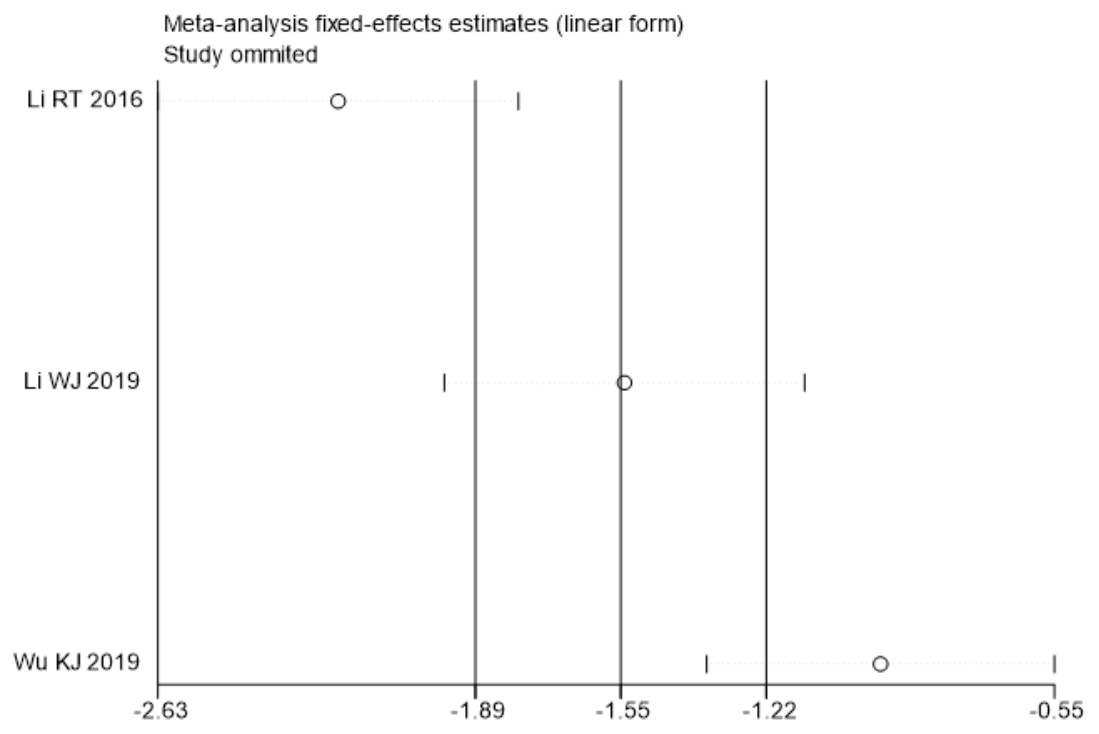

Supplement: Supplementary file 7 [file medi-103-e35832-s007.tif]

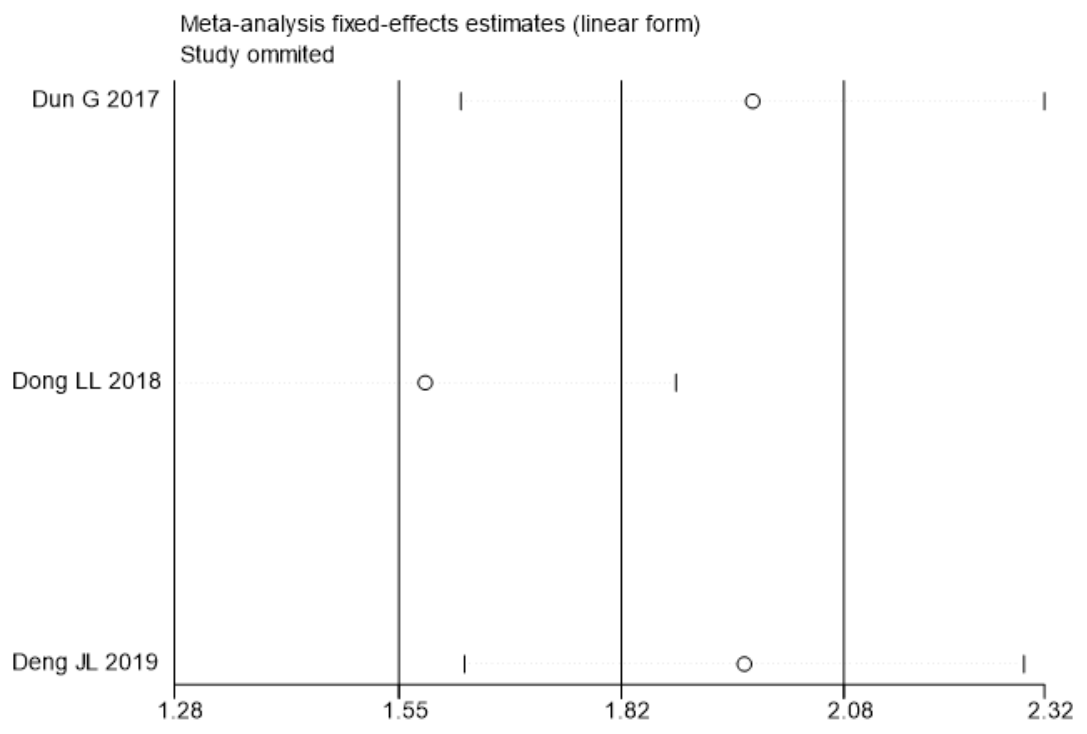

Supplement: Supplementary file 8 [file medi-103-e35832-s008.tif]

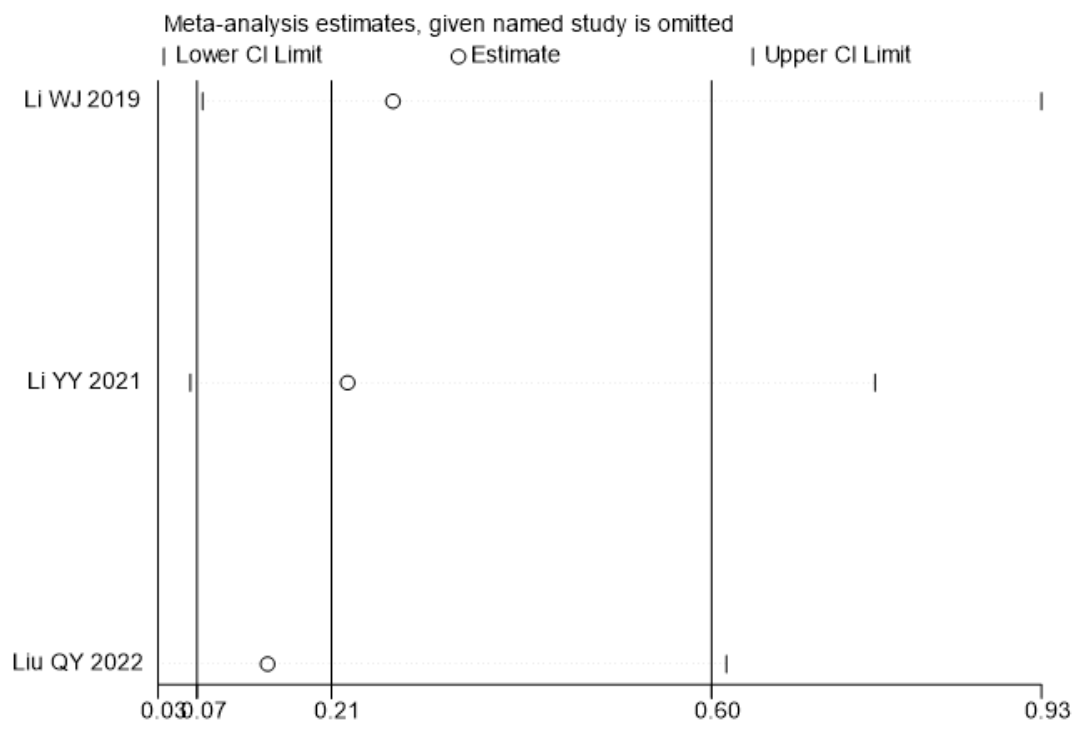

Supplement: Supplementary file 9 [file medi-103-e35832-s009.tif]
